# Lectures on Military Surgery

**Published:** 1861-07

**Authors:** E. Andrews

**Affiliations:** Professor of Surgery; Surgeon to the Mercy Hospital; to the Chicago Med. Dispensary, Etc.


### CHICAGO MEDICAL EXAMINER

N. S. DAVIS, M. D., Editor.---FRANK W. REILLY, M. D., Ass't Editor.

VOL. II.] JULY, 1861. [NO. 7

### Original Contributions.

ARTICLE I.

### LECTURES ON MILITARY SURGERY:

DELIVERED DURING THE SUMMER COURSE OF THE MEDICAL DEPARTMENT OF LIND UNIVERSITY.

By E. ANDREWS, A. M., M. D.,

PROF. OF SURGERY; SURGEON TO THE MERCY HOSPITAL; TO THE CHICAGO MED. DISPENSARY, ETC.

### LECTURE THIRD.

MARCHES.

Troops upon the march usually proceed by public highways in long columns. As time is the very essence of military plans, a greater speed in marching is often equivalent to superiority of force. Hence men are not unfrequently urged over a great distance in a single day, and in these cases a number of them will usually succumb to the heat, dust and fatigue, so far as to require the Surgeon's assistance. For this purpose, the Surgeon should ride in the rear of the column, in order that men who become sick or exhausted may step out of the ranks and wait until the Surgeon comes up. He should then examine them, prescribe for them, and either order them back to the ranks, or assign them a place in the wagons, according as they are or are not able to proceed on foot. Some discretion is to be used in this duty, lest lazy men impose on the humanity of the medical officer, and feign sickness, in order to obtain an opportunity to ride.

If the Surgeon, or any other mounted officer has occasion to ride past the column, he should proceed by the leeward side, in order that the dust raised by the horse may not add to the discomfort of the men.

Some of the men in every long march suffer from sore feet. This generally results from ill-fitting shoes or from sweaty feet. New shoes are particularly liable to hurt the feet. In providing for recruits, therefore, care should be taken that no man has a pair which pinch. Some men are troubled with inordinate sweating of the feet, even in very cool weather. In these cases, the stockings are soon wet, and the cuticle becoming softened and macerated, rubs off, and the feet become terribly sore. The preventive remedy for this evil is simple and effectual: Let the man put an ounce of finely powdered alum in the bottom of each stocking, and he will walk all day, for a week or two together, with perfectly dry and comfortable feet. After awhile, as the effect of the astringent wears off, the sweating may show signs of returning, when a repetition of the same remedy will, as before, effectually suppress it.

In the middle of the day the men are halted for dinner. Care should be taken that this halt is made where good water is obtainable.

At night the troops either "bivouac" or encamp.

The bivouac is simply the usual arrangement of guards, etc., which are adopted in any encampment; but the men lie down without shelter on the open field. This method of repose is adopted whenever the men from any cause are separated from their tents, or when from urgent danger, it is unsafe to have them leave their position on the field to go into camp.

Such circumstances often allow no chance to look after sanitary measures, but if possible, high and dry ground should be chosen, free from malaria and moisture. If the weather is cold, the sheltered side of a hill, or a forest, may be selected, and unless the secrecy of the location is to be preserved, fires should be built. Where parties are forced to bivouac in deep snow, great comfort is obtained by covering the men with snow outside of their blankets, or by burrowing into drifts for a lodging, as was practiced by Dr. Kane in the North.

CAMPS.

At the end of a day's march, the army usually encamp regularly under tents. The spot being properly marked out, the men await the arrival of the tents, which they pitch in regular rows, so as to form streets. While waiting for the tents, they gather wood for fires, and prepare for cooking their supper. Supper being over, they retire to rest. Among volunteer soldiers, great laxity is apt to prevail in these duties. Neither they, nor their officers being aware of the necessity of adhering to the regulations for the sake of health. If the men are exhausted, they are prone to lie down on the grass in wet or sweaty clothes, and fall asleep, neglecting both their supper and their tents. In the night they get chilled, and by morning, have laid the foundation of many cases of diarrhœa, dysentery, and fever. It is the duty of the Surgeon to show the officers the danger of this negligence. The men, in spite of their weariness, should be forced to cook and eat their suppers, and pitch their tents promptly and regularly, and if wet, to change their clothes. They will then sleep soundly, and wake in the morning invigorated for renewed exertions, instead of sickened and prepared for the hospital.

If the camp is to be occupied for a considerable time, some extra care is required. First, the location should be a healthy one. Next, the men should be provided with hay or straw to sleep upon, and this litter must be turned out and aired on every dry day. The tents should each be surrounded with a slight trench, to take off any surface water which may accumulate during rains. A privy must be constructed for the use of the men, in a suitable place. The regulations direct the privy for the men, to be located one hundred and fifty yards in front of the color line, and that of the officers, to be one hundred yards to the rear. Surgeon Tripler, U. S. A., advises instead of this, that both be put always on the side towards which the prevailing winds blow, in order to avoid the effluvia arising from them.

The construction of these temporary sinks is as follows: A trench is dug, about ten or twelve feet in length. At each end a strong forked stake is planted and a heavy pole laid upon them, across which the soldiers sit. The whole is enclosed by placing a thick row of bushes around it. After a few days the trench should be carefully covered up and a new one dug.

Among volunteers great care is required to make the men observe the laws of camp cleanliness. They are prone to scatter portions of their rations among their straw, or throw them upon the ground about the tents, and even to excrete their urine upon the ground in the vicinity, instead of going to the sink prepared for the purpose. Such deposits begin, in a very few days, to contaminate the air of a camp, and to produce an increase of diarrhœa and dysentery.

TENTS.

A variety of tents have been used for military purposes. The most common is the ordinary square ridge-pole tent. This is light, and sufficiently comfortable for summer campaigns. It is rendered more capacious sometimes, by pitching it higher, and adding a perpendicular cloth or curtain from the eaves to the ground. This constitutes the "wall tent."

In cold weather, circular, or rather conical tents are preferable, on account of the less surface which they expose to the cold air. For winter or autumn use, our Government sometimes employ the "Sibley tent." This is constructed after the pattern of the Camanche lodges. It is conical, and being intended to contain a fire, has an opening at the top for the exit of the smoke. The centre pole does not reach the ground, but stands upon the top of an iron triprod, under which the fire is built and to which a kettle may be hung. In winter, a basement is dug about three feet in depth, which protects the men from wind and very much increases their comfort. At the summit of the cone a sort of wind sail or wing is attached for summer use, which serves to direct the current of air for ventilation. These tents, which will accommodate twelve men each, were used by our army in Utah during an entire winter in that severe climate, and were found to render the men perfectly comfortable.

A variety of other tents have been devised for camp life, but the discussion of their merits in full, belongs rather to the military than to the medical officer. A very fine wall tent may be seen at the encampment of the Sturges' Rifles, near this city.

### HUTS.

In cold weather it is often necessary to construct huts for the camp. In timbered regions these may be built precisely like the log cabins of the early settlers. In rocky countries stone huts may be erected; and in any region very warm huts may be made by sinking a pit in the ground and roofing it with turf, boards or tent cloth.

The huts must always be surrounded with ditches to take away the surface water.

### BARRACKS.

Permanent barracks are built of brick, stone or iron, but those for temporary use are often constructed of rough boards.

In planning these edifices, it must be borne in mind that bad ventilation will cause more deaths by sickness than all other errors combined.

To avoid such evils, a very free supply of fresh air should be admitted at or near the floor of the rooms, and the foul air allowed to escape at the top. The apartments should be lofty, and, if possible, should allow the same space per man as a hospital, viz., one thousand cubic feet. In addition to the wards for sleeping, there must be spacious halls and staircases; and lastly, wide verandahs, in which the men can sit protected from the sun, without crowding together in the sleeping rooms.

### HOSPITALS.

The place where the sick and wounded are taken care of, whether within walls of canvas or of hewn stone, is called the Hospital.

The persons connected with the Hospital are—1st, The Surgeons and Assistant-Surgeons; 2d, The Steward; 3d, The Ward Masters; 4th, The Nurses; 5th, The Orderlies and other subordinates.

The senior medical officer of any separate division of the army is called the Medical Director. He is obliged to keep the muster and pay rolls of the attachès of the hospital, including such patients as are transferred to him from other corps. He appoints one medical officer, whose business it is to see that proper supplies are always on hand, and who is called the Medical Purveyor. All requisitions upon the Purveyor are to be approved by the Director. Regular returns are, of course, made of all these things to the authorities above them, and for this purpose blank forms are provided for every report.

The senior medical officer of a regiment or hospital keeps a register of patients, a book for recording cases, an order book, a letter book, and a prescription and diet book. The latter is carried by an attendant on his rounds among the patients, and the directions for medicine and diet entered at once on the spot. At permanent military posts the Surgeon is also obliged to keep a constant meteorological register.

At a regular hour in the day the Surgeon's Call is beaten, when the first sergeants bring forward to the hospital all the sick in their companies, presenting a list. In camp this call is beaten in the morning, and on marches in the evening. The Surgeon examines the patients and orders such as are too sick to remain in quarters, into the hospital. Those who are convalescent or slightly sick are returned to their quarters, and those who are well, and only pretending to be sick, are ordered on duty. A daily report is made to the commanding officer, that it may always be known who and how many are fit for service.

Finally, the medical officer selects the matrons, nurses and cooks for the hospital, and uses his authority to see that they execute their duties faithfully.

The Steward has charge of the material affairs of the hospital.

The Ward Master is one of the nurses, and looks after the bedding, etc.

The nurses are generally taken from the ranks, and one nurse is allowed to every ten patients. From the nature of the case a large part of the nurses must be males; but since the exploits of Florence Nightingale in the Crimean war, female nurses have been admitted in the British army. In the United States the same experiment is being tried. Miss Dix, famed for her influence in originating insane asylums in every part of the United States, has been placed in charge of the appointment of female nurses and of their introduction to their duties. Miss Dix has hitherto remained at Washington and Fortress Monroe, while Mrs. Yates, as her subordinate, has charge of the female nurses at Cairo, in this State.

The requirements of a female nurse are, that she shall be over thirty years of age, shall be sound and vigorous in body, and shall present sufficient testimonials, signed by clergymen and physicians, attesting her moral character and her capacity in her profession.

One cook is allowed for every thirty patients.

The Steward has the pay of an Orderly Sergeant. The Matron has six dollars a month and one ration a day. The cooks and nurses taken from the ranks receive the common pay of soldiers, with twenty-five cents a day extra.

It is probable that the introduction of female nurses in field hospitals will not be attempted, but their employment, under judicious selection and management, must be of great advantage in general hospitals. It is easy to see that the patients may thus receive a more kindly and home-like care than they ordinarily get from their untutored or half tutored comrades.

